# Genome-Wide Study of the Adaptation of *Saccharomyces cerevisiae* to the Early Stages of Wine Fermentation

**DOI:** 10.1371/journal.pone.0074086

**Published:** 2013-09-05

**Authors:** Maite Novo, Ana Mangado, Manuel Quirós, Pilar Morales, Zoel Salvadó, Ramon Gonzalez

**Affiliations:** Instituto de Ciencias de la Vid y del Vino (Consejo Superior de Investigaciones Científicas (CSIC), Universidad de La Rioja, Gobierno de La Rioja), Logroño, Spain; Texas A&M University, United States of America

## Abstract

This work was designed to identify yeast cellular functions specifically affected by the stress factors predominating during the early stages of wine fermentation, and genes required for optimal growth under these conditions. The main experimental method was quantitative fitness analysis by means of competition experiments in continuous culture of whole genome barcoded yeast knockout collections. This methodology allowed the identification of haploinsufficient genes, and homozygous deletions resulting in growth impairment in synthetic must. However, genes identified as haploproficient, or homozygous deletions resulting in fitness advantage, were of little predictive power concerning optimal growth in this medium. The relevance of these functions for enological performance of yeast was assessed in batch cultures with single strains. Previous studies addressing yeast adaptation to winemaking conditions by quantitative fitness analysis were not specifically focused on the proliferative stages. In some instances our results highlight the importance of genes not previously linked to winemaking. In other cases they are complementary to those reported in previous studies concerning, for example, the relevance of some genes involved in vacuolar, peroxisomal, or ribosomal functions. Our results indicate that adaptation to the quickly changing growth conditions during grape must fermentation require the function of different gene sets in different moments of the process. Transport processes and glucose signaling seem to be negatively affected by the stress factors encountered by yeast in synthetic must. Vacuolar activity is important for continued growth during the transition to stationary phase. Finally, reduced biogenesis of peroxisomes also seems to be advantageous. However, in contrast to what was described for later stages, reduced protein synthesis is not advantageous for the early (proliferative) stages of the fermentation process. Finally, we found adenine and lysine to be in short supply for yeast growth in some natural grape musts.

## Introduction

Winemaking is a complex biotechnological process in which yeast cells, most commonly from the species *Saccharomyces cerevisiae*, play the main role by metabolizing grape must sugars into ethanol, carbon dioxide and hundreds of other secondary products. Industrial wine yeast strains are highly specialized organisms which have been selected from spontaneous must fermentations, as well as cellar and vineyard environments, according to specific technological properties [1]. During alcoholic fermentation, yeast cells face stressful conditions mainly characterized by high osmotic pressure (180-260 g/l of sugar content), increasing ethanol content, anaerobiosis and progressive depletion of essential nutrients (nitrogen, vitamins, lipids).

The mechanisms involved in the adaptation of *S. cerevisiae* to the winemaking environment have been studied by both conventional and genome-wide technologies. Among the genome scale approaches, transcriptome analysis is perhaps the method most frequently employed to analyze the interaction between yeast cells and the fermentation environment. Transcriptomic data have provided evidences, at the gene expression level, on most of the adaptation strategies previously described on the basis of physiological studies and individual gene phenotype analysis. Throughout the fermentation process, and in order to adapt to the quickly changing environmental conditions, yeasts sequentially activate or repress different sets of genes involved in several metabolic pathways. After inoculation into grape must, changes at the transcriptome and proteome levels point to the induction of the glycolytic pathway, activation of growth related biosynthetic processes, and carbon catabolite repression [2–5]. As the culture approaches stationary phase, a general stress response is triggered, characterized by induction of the common environmental response (CER), the environmental stress response (ESR), and several heat-shock genes [6–9]. Marks and co-workers [10] identified a group of 223 genes induced at different moments during fermentation and proposed this to be a specific fermentation stress response (FSR). In addition, Rossouw and co-workers [11] correlated metabolite concentrations with the most significant changes in gene expression/regulation all through alcoholic fermentation. They found regulatory changes involving glycolytic metabolites during the initial stages of fermentation. By the end of fermentation, sterol metabolism appears represented, suggesting a role of lipid metabolism in membrane stabilization in the presence of high ethanol concentrations.

These examples illustrate the contribution of transcriptomic studies to highlight the relevance of different nutritional limitations and stress factors for the physiology of yeast in different steps of the fermentation process. However, it has been shown that not all genes relevant for a biological process can be identified by their transcription profile, and conversely, a transcriptional response against a specific environmental condition do not always indicate the relevance of the cognate gene for adaptation to this condition [12,13]. In the same line, Delneri and co-workers [14] concluded that genes that are major controllers of growth rate are not under growth-rate control themselves.

Yeast knock-out (YKO) collections, covering 96% of the yeast genome, are among the most useful tools derived from the international efforts for genome sequencing and functional analysis of *S. cerevisiae* [15]. The inclusion of distinct tags that identify each strain (molecular barcodes) paved the way for the development of chemogenomics in yeast. These collections allow for a genome-wide scale phenotypic characterization of YKO collections by making all strains compete for growth in liquid medium [16]. The output of the competition experiments was originally analyzed by using specific microarrays [15], but high throughput sequencing is now becoming the method of choice [17]. Using this strategy, genes whose activity is required for efficient growth in a given condition (for example under a particular stress) are identified by depletion of the cognate barcodes. Conversely, genes whose deletion improves fitness in a given condition can be identified by an increased relative abundance of the cognate barcodes. The chemogenomic approach involves two types of analysis, Haploinsufficiency profiling (HIP) and Homozygous profiling (HOP), using respectively the heterozygous deletion collection and the homozygous one. Haploinsufficiency occurs when one functional copy of a gene in a diploid organism is insufficient to result in a wild-type phenotype. The HIP analysis is expected to identify, for example, the targets of a stress factor or a drug (used at a semi-permissive level). The HOP analysis is, in turn, expected to identify genes required to overcome the harmful effects of a cytotoxic or stress agent.

The primary attribute that makes the competition experiments suitable for functional genomics is the possibility to screen large numbers of strains, including complete yeast deletion libraries, in a single experiment. In addition, the competition setting is much more sensitive for small fitness differences than individual analysis of growth curves. These advantages of competition experiments can be very well complemented by the use of continuous culture, as demonstrated by Delneri and co-workers [14]. Continuous culture allows increased sensitivity because the competition may continue for a large number of generations under constant conditions. Furthermore, environmental conditions and growth rate are more reproducible under steady state than under batch or sequential batch conditions.

The chemogenomic approach was initially developed to identify targets and resistance mechanisms for small cytotoxic molecules and drugs [15,16]. It has also been used to get insight into a variety of stress resistance mechanisms and other biological questions. In addition, some winemaking related stress factors have been studied using genome scale yeast deletion collections, either by competition experiments or by phenotypic analysis of individual strains. These include osmotic and ethanol stress [18–24], high-sucrose [25], high-glucose [26] and growth on commercial or synthetic grape must [14,27].

The use of BY-series based yeast deletion collections, together with the quickly evolving environmental conditions during the industrial fermentation process and the low number of generations, impose some restrictions in order to design competition conditions relevant to understand yeast adaptation to winemaking. Delneri and co-workers [14] used continuous culture for the analysis of haploinsufficiency by competition experiments, in several nutrient limited media, as well as commercial grape must. While not specifically designed to study wine fermentation, this work showed the suitability of the continuous culture approach to study wine related growth conditions. On the other hand, Piggott and co-workers [27] were able to extend their competition assay during the stationary phase of the fermentation of synthetic must by amplifying the number of viable cells in each sample through an enrichment step in complex medium.

In this work we have established continuous culture conditions mimicking two different stages during wine fermentation. Phase I corresponds to high growth rate conditions and is characterized by high sugar and nitrogen content and a negligible ethanol concentration. Phase II corresponds to the transition to stationary phase, when nitrogen sources become limiting. These two different growth conditions were maintained for 10 or 20 generations, depending on the specific yeast deletion collection (homozygous or heterozygous, respectively). This experimental set up resembles that of Delneri and co-workers [14], in so far as it is based on continuous culture, but is different from it in several aspects (table S1), notably sugar concentration, oxygen availability, and the fact that only the heterozygous collection was analyzed in that work. On the other hand, it is complementary to the work by Piggott and co-workers [27], since the continuous culture set up allowed us to focus on the proliferative stages of the fermentation; while the timing of the experiment and sample treatment made the experiment by Piggott an co-workers [27] more informative about latter stages of the process. In addition to this complementarity, focusing on yeast growth during winemaking was justified by the fact that biomass production is a key step in winemaking, determining the global kinetics of the fermentation [28].

## Materials and Methods

### Yeast strains and collections

*S. cerevisiae* BY4743 (*MATa*/*MATα his3Δ1/his3Δ1 leu2Δ0*/*leu2Δ0 met15Δ0*/*MET15 LYS2*/*lys2Δ0 ura3Δ0*/*ura3Δ0*) was used for batch characterization, and as a reference for the phenotypic characterization of deleted strains. The homozygous and heterozygous yeast deletion strains, in the diploid BY4743 background, were purchased from Open Biosystems (Huntsville, USA). Balanced pools for each collection were prepared on YPD broth (2% glucose, 2% peptone, 1% yeast extract) containing 200 µg/mL G418 (Sigma-Aldrich) and 15% glycerol, following the protocol described by Pierce and co-workers [29]. One mL aliquots of the yeast deletion strain pools were prepared in 2 mL screw-capped criotubes (Sarstedt, Nümbrecht, Germany) and stored at -80 °C.

### Batch characterization

Both batch and continuous cultures were run in a DASGIP parallel fermentation system (DASGIP AG, Jülich, Germany) equipped with four SR0400SS vessels. In all cases, agitation was maintained at 100 rpm, and the temperature kept at 28 °C using a water bath. Anaerobic conditions were maintained by gassing the headspace of the bioreactors with pure nitrogen (0.5 L min^-1^). The off-gas was conducted through cooled condensers (1-3 °C) and the concentration of CO_2_ in the exhaust gas recorded every 30 s with a GA4 gas analyzer (DASGIP AG).

Batch cultures using *S. cerevisiae* strain BY4743 were performed in duplicate in 200 mL working volume in order to determine relevant physiological parameters (μ_max_ and specific consumption/production rates for glucose, fructose, ethanol, CO_2_ and biomass) during the fermentation of synthetic grape must. For that purpose, strain BY4743 was grown in YPD broth for 48 h and 28 °C and inoculated to a final OD_600_ of 0.2. For these experiments a modification of a previously described synthetic must was used [30]. The medium contained the following components per liter: glucose, 100 g; fructose, 100 g; malic acid, 6 g; citric acid, 6 g; Yeast Nitrogen Base w/o amino acids and (NH_4_)_2_SO_4_, 1,7 g; NH_4_Cl, 306 mg; alanine, 97 mg; arginine, 245 mg; aspartic acid, 29 mg; cysteine, 14 mg; glutamic acid, 80 mg; glutamine, 333 mg; glycine, 12 mg; histidine, 23 mg; isoleucine, 22 mg; leucine, 32 mg; lysine, 11 mg; methionine, 21 mg; phenylalanine, 25 mg; proline, 400 mg; serine, 52 mg; threonine, 50 mg; tryptophan, 116 mg; tyrosine, 13 mg; uridine, 83 mg, and valine, 29 mg. A mix of ergosterol (15 mg), sodium oleate (5 mg) and Tween 80 (0.5 mL) was used as anaerobic factors. The pH of the medium was maintained at 3.5 by the automated addition of 2N NaOH.

The physiological characterization of batch fermentations allowed the definition of different phases. Phase I would correspond to yeast exponential growth phase, while Phase II would correspond to the transition to stationary phase. In this last stage nitrogen sources become limiting but ethanol concentration is still below 3%.

### Competition experiments in continuous culture

Competition experiments of the genome-wide collections of mutants were performed in triplicate using conditions that mimicked Phases I or II of a batch fermentation (equivalent to around 14 and 22 hours after inoculation of a batch fermentation, respectively). For such purpose, different feed formulations were used. The feed employed for mimicking Phase I was identical to the medium previously used for batch characterization. In order to mimic Phase II, NH_4_Cl was removed from the medium and the concentration of each amino acid reduced to 70% of the original one. Final concentration of histidine, leucine and uridine was adjusted to 200 mg/L, 600 mg/L and 283 mg/L, respectively, as suggested by Harsch and co-workers [31].

One mL of either the homozygote or heterozygote pool stored at -80 ^°^C was inoculated into 50 mL of YPD broth supplemented with 200 µg/mL G418 and incubated overnight at 28 °C and 150 rpm. Bioreactors containing 200 mL of the previously described synthetic must were inoculated with this pre-culture to an initial OD_600_ of 0.2. Cultures were grown in batch mode during about 12 h prior to triggering the continuous cultures. According to the data obtained from batch characterization, dilution rate was set to 0.23 h^-1^ for competitions mimicking Phase I and to 0.04 h^-1^ for those mimicking Phase II. Competition experiments using the heterozygous collection were run for 20 generations (corresponding to 60 h for Phase I or 347 h for Phase II) while those performed with the homozygous collection were run for 10 generations (30 h for Phase I or 174 h for Phase II). Yeast cell samples were taken at the onset of the continuous cultures, and after the indicated numbers of generations, in order to compare pool compositions at the beginning and the end of the competition experiments. Additional triplicate competition experiments, using YPD broth (D=0.23 h^-1^), were performed in order to differentiate deletions unspecifically affecting yeast growth from those specific for Phase I or Phase II fermentation conditions (see below).

### Fitness analysis

Samples corresponding to 2 OD_600_ of cells were used for the genomic DNA extraction using the YeaStar^TM^ Genomic DNA Kit (Zymo Research). DNA concentration was measured using a Nanodrop spectrophotometer (Thermo Scientific). Amplification of the barcodes (uptags and downtags), hybridization, and scanning were performed according to the protocol described elsewhere [29]. Slight modifications concerning the hybridization of Tag4 microarrays by using reagents and protocols from the GeneChip Hybridization, Wash and Stain Kit (Affymetrix) were made. The data discussed in this publication have been deposited in NCBI’s Gene Expression Omnibus [32] and are accessible through GEO Series accession number GSE46145 (http://www.ncbi.nlm.nih.gov/geo/query/acc.cgi?acc=GSE46145.

Hybridization data were used to obtain fitness ratios and p-values by using the scripts developed by Pierce and co-workers [29] (available at http://chemogenomics.stanford.edu/supplements/04tag/download). Values for the ratios between tags at time 0 and after 10 or 20 generations were used for the identification of homozygous deletions and haploinsufficient genes conferring growth defect in synthetic must, respectively. The inverse ratios were calculated for the identification of haploproficient genes and genes whose complete deletion confers growth advantage in the competition conditions. Genes lists were elaborated by including genes showing log_2_ fold changes >1 and p values <0.05. However genes showing a log_2_ fold changes >0.58 in the parallel (direct or inverse) analysis in YPD for the same number of generations were excluded.

### Enological assessment of individual strains

Selected strains were subjected to phenotypic characterization in synthetic must (see media composition for batch cultures above) in order to assess the enological relevance of the genes identified by fitness analysis. On one side, growth kinetics profiles were obtained by growing cells in 16 mm internal diameter tubes, filled with 7 mL of synthetic must, recording increase in turbidity with a 2100N Turbidimeter (HASH, Loveland. CO). Pre-cultures were grown in YPD for 48 h, using 100 μL to inoculate each culture tube. Kinetic curves were run in fully independent triplicates, using three independent pre-cultures for inoculation. Relative biomass of the test strain at time 24 h, compared to the control strain (BY4743) grown in the same experimental batch, was taken as the first parameter to assess the enological relevance of each deletion.

On the other side, microfermentation experiments were also run in fully independent triplicates. For that purpose, 15 mL of the same synthetic must were dispensed in 50 mL Falcon tubes and capped with fermentation locks filled with mineral oil. Three independent inocula were prepared as described above. Fermentation kinetics was monitored by daily recording weight loss. Fermentations were stopped after 21 days. Depending on the experiment, residual sugar at this point ranged from 1 to 3.5% for the wild type, and up to 8% for severely impaired strains. Tubes were homogenized and sampled in order to measure OD_600_ as a second parameter for enological relevance of the deletion. Final OD_600_ values for BY4743 varied from 4.7 to 5.2, depending on the experiment.

Additionally, for the phenotypic analysis of several homozygous auxotrophic mutants, supplementary fermentations were performed in a natural Malvasía/Viura must containing approximately 22% fermentable sugars and supplemented with histidine, leucine and uridine to a final concentration of 200 mg/L, 600 mg/L and 283 mg/L, respectively. In addition adenine (40 mg/L), inositol (73 mg/L), lysine (30 mg/L) or serine (375 mg/L) were supplemented for specific fermentation experiments as indicated below.

One way analysis of variance was carried out by means of IBM SPSS Statistics v. 20 program. Means were compared using DMS, with significance level at 5%.

## Results

Yeast deletion mutants specifically and significantly showing growth advantage or disadvantage in synthetic must under Phase I (exponential growth phase) or Phase II (transition to stationary phase) fermentation conditions were identified by fitness analysis of the competition experiments as described above. Briefly, Phase I growth conditions were emulated in continuous culture at a dilution rate of 0.23 h^-1^ using as feed the same synthetic must formulation as used in batch cultures. Continuous culture conditions used to emulate Phase II involved a change in feed composition (same sugar content as synthetic must, but reformulated nitrogen content; see above) and a reduced dilution rate (0.04 h^-1^). In both cases, these growth conditions were maintained for 10 generations for HOP analyses and 20 generations for HIP analyses. After DNA extraction and microarray hybridization values for the ratios between tags at time 0 and after the indicated numbers of generations were used for the identification of gene deletions conferring growth defect in synthetic must. We will refer to these results as direct HIP or HOP analyses. The inverse ratios were calculated for the identification of deletions conferring growth advantage in synthetic must. These results will be referred to as inverse HIP or HOP analysis. Genes lists were elaborated by including genes showing log_2_ fold changes >1 and p values <0.05. However genes showing log_2_ fold changes >0.58 in the parallel (direct or inverse) analysis in YPD for the same number of generations were excluded. The final gene lists are shown as supplementary material (Workbook S1). GO enrichment analysis for these gene lists was performed by using YeastMine tools [33]. Only GO terms with enrichment p-values < 0.01 were considered for discussion. In order to facilitate the interpretation gene groups were constructed by including genes from GO terms sharing more than 2/3 of the genes with other categories or preexisting groups. In most cases we did not find a single GO category including all genes in each group. Furthermore, for similar GO terms that additionally share gene number and p-value, a single definition, corresponding to the first GO number is shown for illustration. The rest of the GO terms are just indicated by their number. Unedited results of the GO enrichment analysis are shown in Workbook S2. Consistency of the experimental data was confirmed independently for the HIP and the HOP analyses by principal component analysis using MIDAS [34]. Data for the same time point and medium (Phase I, Phase II, or YPD), formed discrete non-overlapping groups (data not shown). This consistent set of data was then used to identify genes relevant for fitness under conditions mimicking Phase I or Phase II of alcoholic fermentation, according to the criteria mentioned in Materials and Methods. The eight possible combinations for HIP or HOP analysis, Phase I or Phase II of fermentation, and direct or inverse analysis gave rise to the eight gene lists shown in Workbook S1.

### Direct HIP analysis: Identification of haploinsufficient genes

Our direct HIP analysis revealed 158 different strains showing fitness deficiency under Phase I conditions. Almost one third of them (50 genes) was associated with the single GO term "localization", and related ones (Table 1). Most of them (42 genes) are related to transport processes, with molecular functions as diverse as glucose transport, vesicular transport, metal transport, glutamente export, nuclear pore constituents, proton pumps, protein secretion, mitochondrial protein import or autophagy. If osmotic stress is considered as the main stress factor in Phase I, this would point to these processes as targets for osmotic stress during the first stages of alcoholic fermentation, as well as being important for growth under these conditions. GO terms related to purine metabolism involve 11 genes. Other enriched terms are related to DNA metabolism. Also worth mentioning are the genes *RGT2* and *SNF3*, involved in glucose sensing in *S. cerevisiae*. On the other hand, among the 107 genes showing haploinsufficiency in Phase II, 12 of them are related to lipid metabolic processes, and two additional genes to “lipid homeostasis” (Table 2).

**Table 1:** Gene Ontology enrichment analysis for genes identified by direct HIP analysis under Phase I fermentation conditions.

**GO Term**	**p-value**	**#^a^**	**Genes in group^b^**
localization [GO:0051179]	0.00061	50	ADY2, ARC40, ATG29, ATP19, AVT6, BSP1, CSR2, EAR1, FOB1, FRE4, GEA1, GTR2, INP51, INP54, KAP120, KIP2, MGR2, MIM2, MST27, NPA3, PDR12, PEX25, PEX3, PHB1, PMA1, POM152, PUF4, RAV1, RGT2, ROK1, RVS161, SEC17, SEC62, SGF29, SIT1, SKM1, SKY1, SNF3, SNQ2, SRP1, SSA4, SSD1, SVP26, SWH1, SYH1, SYT1, TRS120, UBP3, YAR1, ZUO1
cellular localization [GO:0051641]	0.00362	32	s
ARF protein signal transduction [GO:0032011]	0.00538	2	s
protein import [GO:0017038]	0.00684	8	s
macromolecule localization [GO:0033036]	0.00731	29	s
transport [GO:0006810]	0.00743	42	s
nuclear import [GO:0051170]	0.00955	5	s
protein localization [GO:0008104]	0.0096	25	s
DNA strand renaturation [GO:0000733]	0.00068	3	HRQ1, MGM101, RAD52
detection of chemical stimulus [GO:0009593/0009730/0009732/0034287/0051594/051606]	0.00167	2	RGT2, SNF3
phosphatidylinositol dephosphorylation [GO:0046856]	0.00318	3	INP51, INP54, TCB3
phospholipid dephosphorylation [GO:0046839]	0.00397	3	S
GMP biosynthetic process [GO:0006177]	0.00538	2	ATP19, GCD11, GUA1, HPT1, ISN1, MEF1, NFS1, PDR12, PMA1, SNQ2, TRS120
purine nucleoside metabolic process [GO:0042278/0046128]	0.00626	11	s
purine ribonucleoside salvage [GO:0006166/0043174/0046037]	0.00794	2	s
DNA replication-dependent nucleosome assembly [GO:0006335]	0.00538	2	CAC2, RLF2
DNA replication-dependent nucleosome organization [GO:0034723]	0.00538	2	s

Unedited results of the GO enrichment analysis are shown in Workbook S2.

a: number of genes shared with its group

b: s = same as above

**Table 2:** Gene Ontology enrichment analysis for genes identified by direct HIP analysis under Phase II fermentation conditions.

**GO Term**	**p-value**	**#^a^**	**Genes in group^b^**
beta-glucan metabolic process [GO:0051273]	0.00152	3	GAS5, PSK1, TRS65
phospholipid metabolic process [GO:0006644]	0.00163	8	ALG8, DAP1, FIG4, FMP30, IDI1, MUM3, OSH6, PAH1, PCT1, PGS1, PLB3, YOR022C
lipid metabolic process [GO:0006629]	0.00334	12	s
response to methylmercury [GO:0051597/0071406]	0.00494	2	HRT3, YLR224W
cellular response to methylmercury [GO:0071406]	0.00494	2	s
lipid homeostasis [GO:0055088]	0.00494	2	GEM1, OSH6
pyridoxal phosphate metabolic process [GO:0042822/0042823]	0.00652	2	BUD16, SNZ2

Unedited results of the GO enrichment analysis are shown in Workbook S2.

a: number of genes shared with its group

b: s = same as above

**Figure 1:**
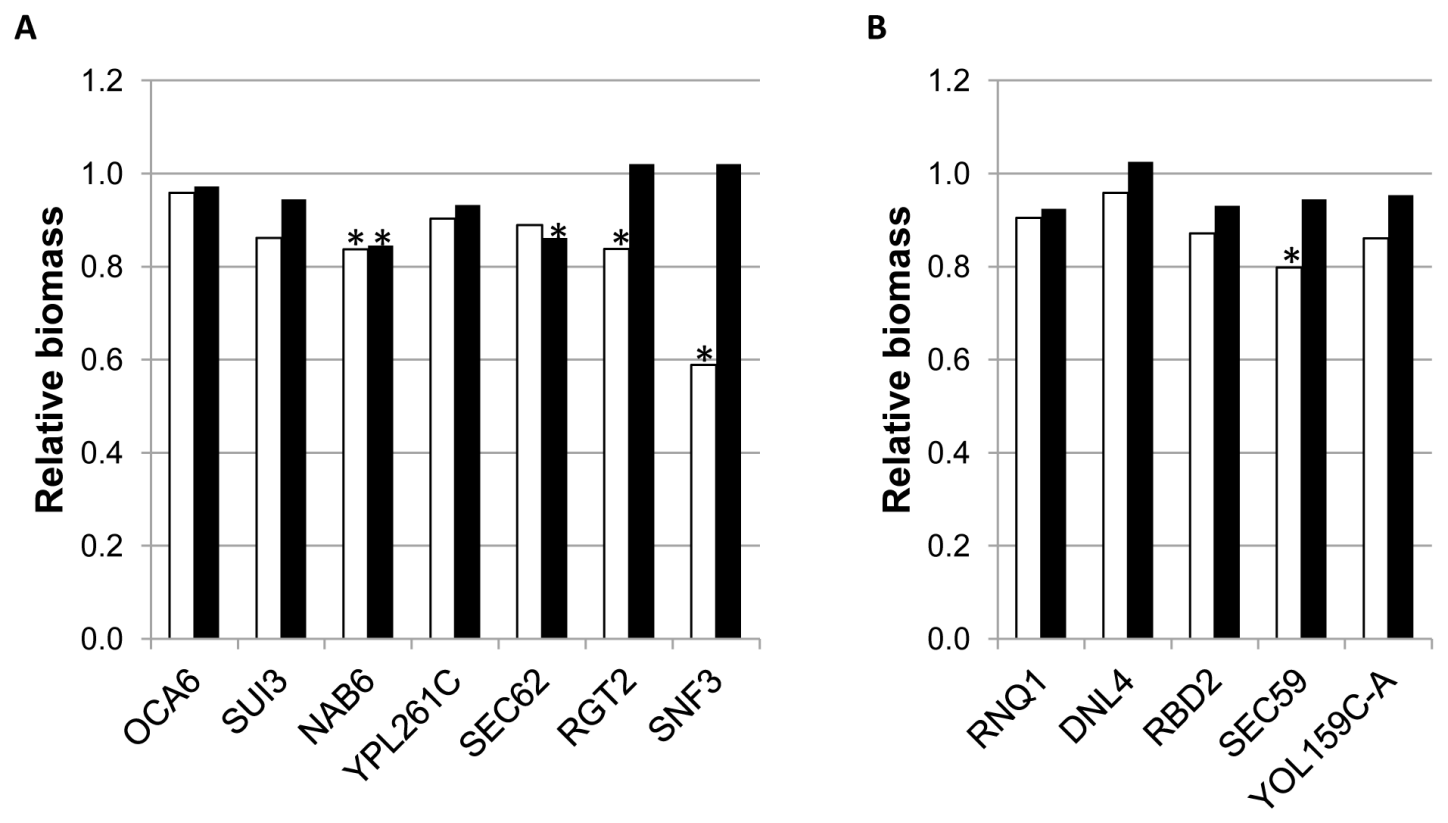
Phenotypic characterization of strains selected by direct HIP analysis under Phase I or Phase II conditions. Detailed legend: Relative biomass after 24 h (white bars) or after 21 days (black bars) for strains identified by direct HIP analysis under Phase I (Panel **A**) or Phase II (Panel **B**) conditions. Relative biomass after 24 h was estimated by comparing turbidity of the indicated strains with the control strain (BY4743) in the same batch. It was measured as NTUs with a 2100N Turbidimeter (HASH, Loveland. CO). Relative biomass after 21 days was estimated by measuring OD_600_ of the homogenized culture and comparing data from the deleted strains with those of the control strain (BY4743) in the same batch. Stars indicate statistically significant differences between BY4743 and the deleted strain.

In order to assess the enological relevance of the cell functions identified in this analysis, the five strains showing the largest log_2_ fold changes, for both Phase I (*OCA6*, *SUI3*, *NAB6*, *YPL261C* and SEC*62*) and Phase II (*RNQ1*, *DNL4*, *RBD2*, SEC*59* and *YOL159C-A*), as well as *RGT2* and *SNF3*, were phenotypically characterized, for short and long term growth on synthetic must (24 hours and 21 days respectively). The general trend was for a slight to severe impairment for growth on synthetic must after 24 hours, although only three strains from Phase I (*NAB2, RGT2*, and *SNF3*) and one strain from Phase II (*SEC59*) showed statistically significant differences with BY4743. In general the effect of growth impairment was mitigated by the end of the fermentation (black bars in Figure 1).

Only seven strains, those deleted for *PGS1, RNQ1, YPL102C, FMP30, DAP1, RBD2*, and *YHR086W-A*, were present in both lists (Phase I and the Phase II) (Figure S1). Functional enrichment analysis did not identify a common theme for these seven genes. Phenotypic characterization showed the same trend as above, moderate growth impairment for 24 hours for almost all the strains (even though not statistically significant), and several strains showing wild type biomass values by the end of the fermentation (Figure S2).

### Direct HOP analysis: Homozygous deletions conferring growth defect in synthetic must

**Figure 2:**
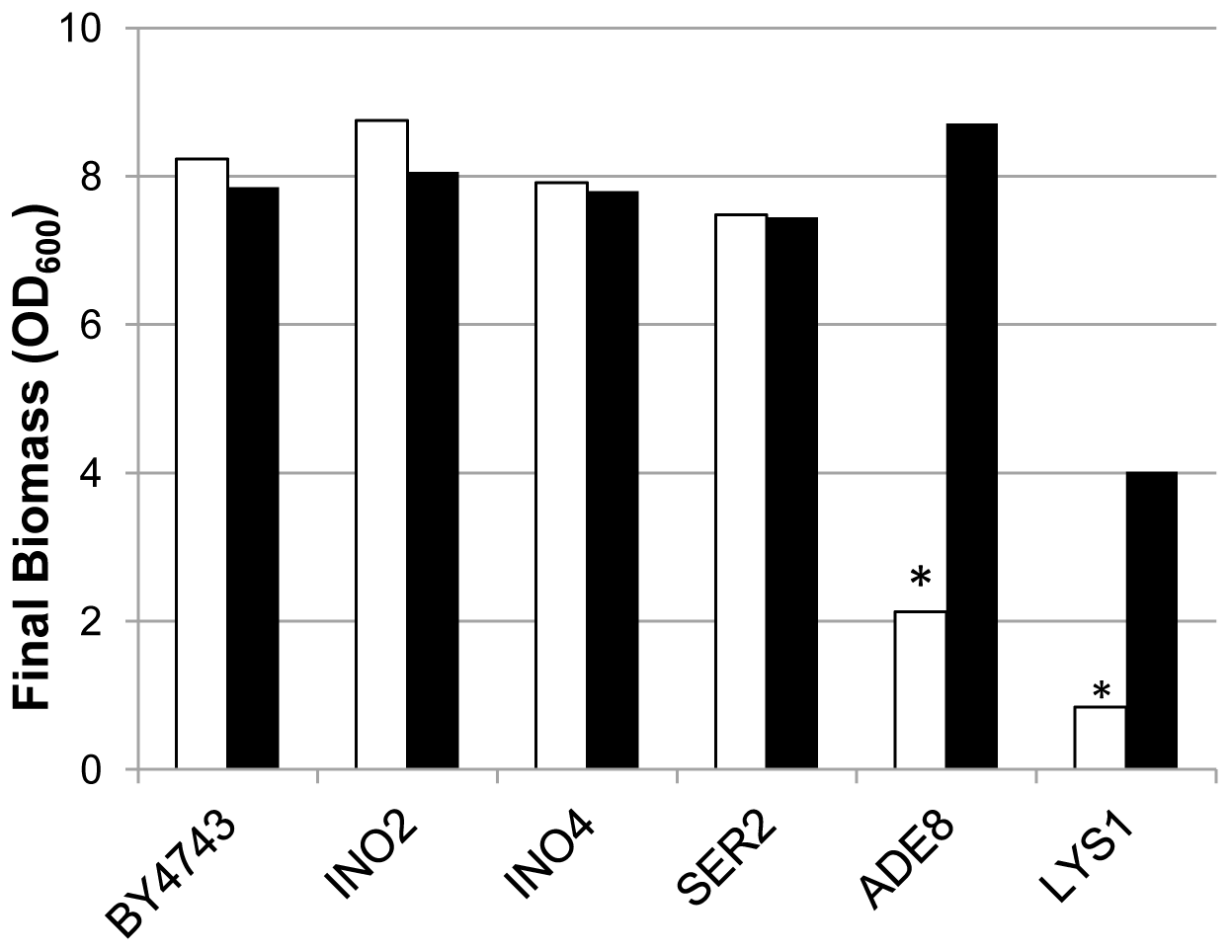
Final biomass of BY4743 and auxotrophic strains fermenting natural grape must. Detailed legend: Final biomass for *S*. *cerevisiae* strain BY4743 and homozygous strains deleted for genes conferring auxotrophy to inositol, serine, adenine and lysine after fermentation on natural Malvasía/Viura grape must (white bars) and the same must supplemented with the specific compound for auxotrophy complementation (black bars).

The list of 83 genes showing fitness deficiency under Phase I fermentation conditions, when deleted in homozygosis, includes several genes whose loss of function had previously been shown to confer auxotrophy to serine, adenine, lysine, or other nitrogen compounds, as well as to myo-inositol. Although these compounds are included in the synthetic must used in the competition experiments, some of them might have been in short supply. The impact of these auxotrophies on the outcome of the fitness analysis was then assessed by analyzing the growth of some of these strains in synthetic must further supplemented, or not, with the appropriate requirements for each strain. As expected, biomass production in the original medium was lower for all the mutants assayed than for the BY4743 wild type strain (Table 3). Supplementation with the required nutrients restored growth to different extents, depending on the particular gene deleted, thus indicating that partial starvation for some auxotrophic requirements might be involved in the fitness defect observed for these strains in synthetic must under Phase I conditions. A similar experiment in natural white grape must confirmed adenine and lysine availability to be limiting for auxotrophic strains (Figure 2). In the case of lysine the phenotype might be also influenced by the BY4743 genetic background (*LYS2*/*lys2Δ0*). On the other side, inositol and serine deficiencies seem to be specific for synthetic must.

**Table 3:** Final biomass values after fermentation of synthetic must with and without additional supplementation.

Deletion	OD max	OD max(with supplement)
None (BY4743)	3,29	N/A
ADE8	0,00	3,16
INO2	1,97	3,74
INO4	2,09	3,78
LYS1	1,38	2,45
SER2	2,87	3,25

Since auxotrophic mutations induced a strong bias in the functional enrichment analysis, thirteen genes, showing clear relation with auxotrophic phenotypes, were removed from the output of the Phase I direct HOP analysis. The enrichment analysis for the remaining 70 genes is shown in Table 4. Genes highlighted by this analysis mainly function in chromatin dynamics and transcriptional regulation.

**Table 4:** Gene Ontology enrichment analysis for genes identified by direct HOP analysis under Phase I fermentation conditions.

**GO Term**	**p-value**	**#^a^**	**Genes in group^b^**
regulation of transcription from RNA polymerase III promoter [GO:0006359]	6.01E-05	3	CKB1, CKB2, SCH9
regulation of transcription from RNA polymerase I promoter [GO:0006356]	0.0037	3	s
transcription from RNA polymerase III promoter [GO:0006383]	0.00778	3	s
DNA replication-dependent nucleosome assembly [GO:0006335/0034723]	0.00107	2	HPC2, NOT3, RLF2, RTT106, SNF2, TOP1
nucleosome assembly [GO:0006334]	0.00149	3	s
DNA replication-independent nucleosome assembly [GO:0006336]	0.0016	2	s
DNA packaging [GO:0006323]	0.0033	4	s
DNA replication-independent nucleosome organization [GO:0034724]	0.00376	2	s
nucleosome organization [GO:0034728]	0.00522	4	s
chromatin assembly or disassembly [GO:0006333]	0.0055	4	s
transcription elongation from RNA polymerase II promoter [GO:0006368]	0.0058	4	s
chromatin assembly [GO:0031497]	0.00835	3	s
DNA-dependent transcription, elongation [GO:0006354]	0.00853	4	s
DNA conformation change [GO:0071103]	0.00932	4	s
cellular response to amino acid starvation [GO:0034198]	0.00222	2	NPR2, SNF2
tubulin complex assembly [GO:0007021]	0.00466	2	PAC10, YEK2
tubulin complex biogenesis [GO:0072668]	0.00566	2	s
regulation of cell differentiation [GO:0045595]	0.00566	2	MDS3, SNF2

Unedited results of the GO enrichment analysis are shown in Workbook S2.

a: number of genes shared with its group

b: s = same as above

**Figure 3:**
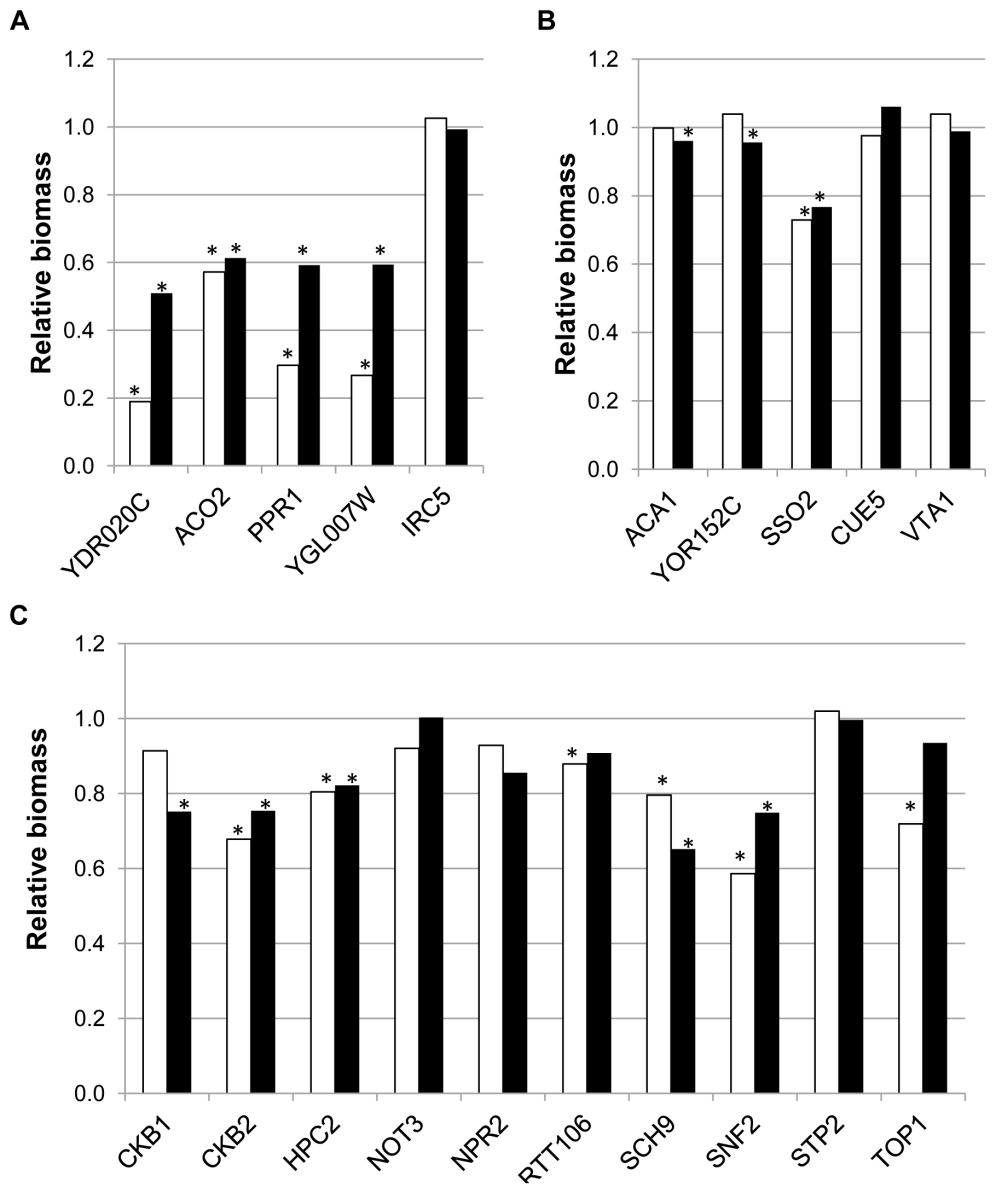
Phenotypic characterization of strains selected by direct HOP analysis under Phase I or Phase II conditions. Detailed legend: Relative biomass after 24 h (white bars) or after 21 days (black bars) of strains selected from the top of the direct HOP analysis list under Phase I (Panel **A**) or Phase II (Panel **B**) conditions; and strains highlighted in the enrichment analysis for HOP experiments under Phase I conditions shown in table 4 (Panel **C**). Relative biomass after 24 h was estimated by comparing turbidity of the indicated strains with the control strain (BY4743) in the same batch. It was measured as NTUs with a 2100N Turbidimeter (HASH, Loveland. CO). Relative biomass after 21 days was estimated by measuring OD_600_ of the homogenized culture and comparing data from the deleted strains with those of the control strain (BY4743) in the same batch. Stars indicate statistically significant differences between BY4743 and the deleted strain.

Some strains showing the largest log_2_ fold changes in the direct HOP analysis for Phase I (Workbook S1), as well as several of the strains highlighted in the enrichment analysis shown in Table 4 (Figure 3, panels A and C, respectively) were selected to assess the enological relevance of the corresponding genes. Apart from *IRC5*, which behaved as the wild type strain in this assay, and should be considered as a false positive, growth in synthetic must was greatly impaired for all the other strains in Figure 3, panel A. Growth impairment is less pronounced for these strains than seen for the strains above, and the number of them showing statistically significant differences with BY4743 is also lower (Figure 3, panel C). These different degrees of impairment in the batch culture assay roughly agree with the different fitness reduction observed for the strains included in each panel (Workbook S1).

Under Phase II conditions 139 genes were identified by direct HOP analysis. GO terms enriched for this data set are shown in Table 5. The most relevant term is "protein folding in endoplasmic reticulum", and this might be related to this molecular machinery being required to cope with the increasing ethanol stress in PhaseII, in order to be able to synthesize functional membrane and cell wall proteins. In addition "localization" and related terms define a pool of 42 genes shared between most of the relevant categories identified in this analysis. However, there is little relationship between this group of genes and those identified in the direct HIP analysis in Phase I conditions also labeled as "localization". In this case, an enrichment in vacuole related functions can be appreciated (autophagy and vacuolar protein sorting). Indeed, in spite of the large number of genes in each one, there are only two genes, *PDR12* and *SYH1*, shared between those labeled with the GO term localization in Phase I for direct HIP analysis and Phase II for direct HOP analysis.

**Table 5:** Gene Ontology enrichment analysis for genes identified by direct HOP analysis under Phase II fermentation conditions.

**GO Term**	**p-value**	**#^a^**	**Genes in group^b^**
protein folding in endoplasmic reticulum [GO:0034975]	1.10E-06	5	DUR1,2, EGD2, EMC1, EMC3, EMC5, EUG1, GSF2, HSP104, JEM1, JID1, SSA1
protein folding [GO:0006457]	1.29E-05	11	s
vacuolar transport [GO:0007034]	0.00045	10	ABP1, ADP1, APS3, ATG22, ATG27, ATG8, ATG9, BLS1, CNL1, EGD2, ERV15, FET4, GSF2, HSP104, IKI3, LDB18, MFT1, MRS4, PDR12, RAS2, RPS18B, SBH2, SMF3, SSA1, SSO1, SSO2, SSU1, SYH1, TPO2, TPO3, TRS33, VPS24, VPS36, VPS62, VPS68, VTA1, WHI3, YAP1802, YPT10, YVC1, ZRT2, ZRT3
localization [GO:0051179]	0.00084	42	s
transition metal ion transport [GO:0000041]	0.00109	5	s
autophagic vacuole assembly [GO:0000045]	0.00139	3	s
protein targeting to vacuole [GO:0006623/0072666]	0.00172	7	s
protein localization to vacuole [GO:0072665]	0.00184	7	s
protein localization [GO:0008104]	0.00282	23	s
metal ion transport [GO:0030001]	0.00343	6	s
establishment of localization [GO:0051234]	0.00489	37	s
gene silencing involved in chronological cell aging [GO:0010978/0031047]	0.00111	2	ACA1, CAF130, DST1, HDA2, HDA3, HIR2, HIR3, IKI3, ISW2, PRR1, RAS2, RDS2, SWP82, USV1, VPS36, WHI5
carbon catabolite regulation of transcription [GO:0045990]	0.00447	4	s
DNA replication-independent nucleosome assembly [GO:0006336]	0.00535	2	s
negative regulation of transcription from RNA polymerase II promoter [GO:0000122]	0.00817	7	s
carbon catabolite activation of transcription [GO:0045991/0006357]	0.00957	3	s
ascospore-type prospore assembly [GO:0031321]	0.00275	3	SPO19, SSO1, SSO2

Unedited results of the GO enrichment analysis are shown in Workbook S2.

a: number of genes shared with its group

b: s = same as above

Also enriched in the Phase II direct HOP analysis is "transcriptional repression", especially in connection with carbon catabolite repression. Some of these strains (among those showing the largest log_2_ fold changes) were also characterized for enological relevance (Figure 3, panel B). In contrast to strains identified under Phase I conditions, only one of them, *SSO2*, could be demonstrated to be actually impaired for short term (24 h) growth in synthetic must.

Four strains were simultaneously identified by direct HOP analysis of Phase I and Phase II. One is deleted for a dubious open reading frame, while the other deletions affect genes involved in protein metabolism and turnover. Deletion of *GSF2*, involved in glucose signaling, results in the greatest impairment for growth in synthetic must among these four gene deletions, and the only statistically significant one (Figure S3).

### Inverse HIP analaysis: Identification of haploproficient genes

Table S2 shows GO enrichment for genes showing haploproficiency under Phase I conditions (79 genes, Workbook S1). According to GO labeling, five groups could be distinguished, with three of them sharing three genes, *PAF1*, *CTR9*, and *HTL1*. GO terms in different groups are also related among them, with mitosis and chromatin organization (modification) appearing as common themes. Products of *PAF1* and *CTR9* participate in the Paf1 complex, responsible for full expression of a subset of yeast genes by modulating the activity of RNA polymerases I and II. Deletion mutants in components of this complex show pleiotropic phenotypes (Betz et al., 2002). *HTL1* is a component of the RSC chromatin remodeling complex, also related to transcriptional control among other chromosome-related functions. These three strains were selected for phenotypic characterization, among others.

**Figure 4:**
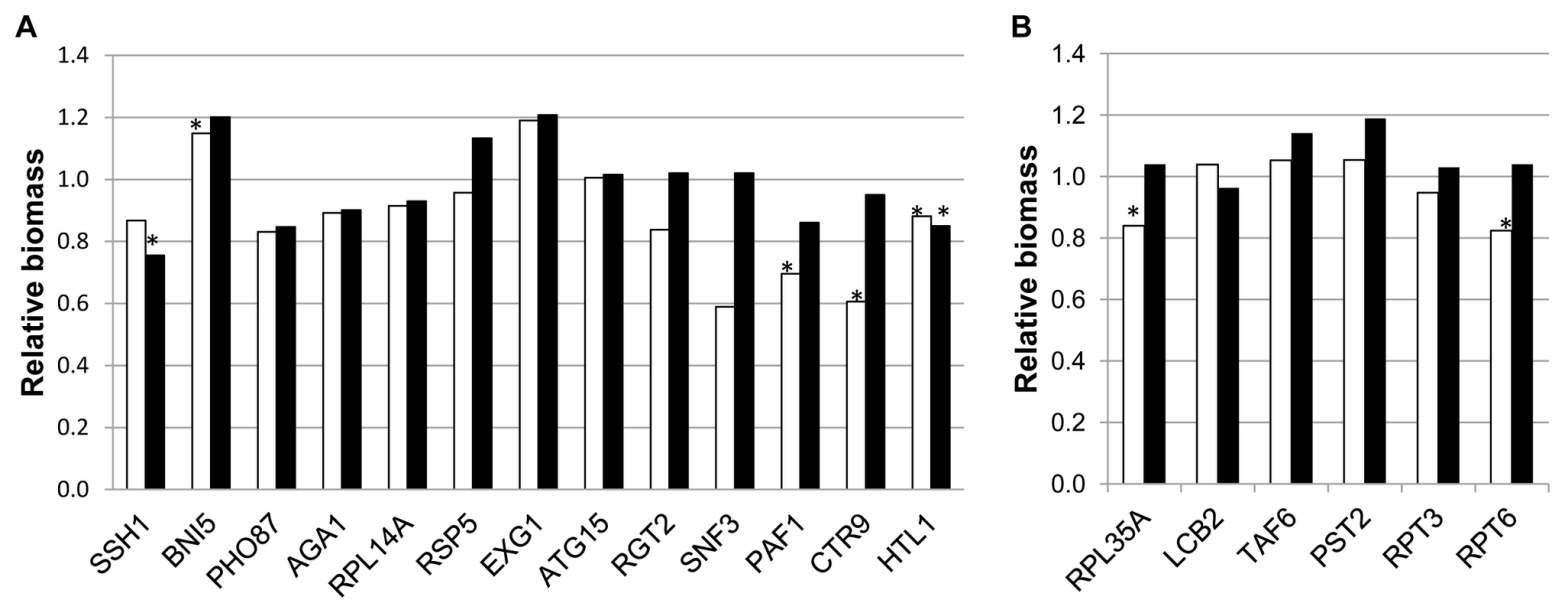
Phenotypic characterization of strains selected by inverse HIP analysis under Phase I or Phase II conditions. Detailed legend: Relative biomass after 24 h (white bars) or after 21 days (black bars) for strains identified in the inverse HIP analysis during Phase I (Panel **A**) and Phase II (Panel **B**) fermentation conditions. Relative biomass after 24 h was estimated by comparing turbidity of the indicated strains with the control strain (BY4743) in the same batch. It was measured as NTUs with a 2100N Turbidimeter (HASH, Loveland. CO). Relative biomass after 21 days was estimated by measuring OD_600_ of the homogenized culture and comparing data from the deleted strains with those of the control strain (BY4743) in the same batch. Stars indicate statistically significant differences between BY4743 and the deleted strain.

Two strains, deleted for one allele of *BNI5* or *EXG1*, showed improved growth in synthetic must (both at 24 hours and as final OD in fermentation experiments). *BNI5* is involved in organization of septins at the mother-bud neck, and appears in one of the main gene groups in Table S2, related to cell cycle. *EXG1* is involved in cell wall assembly and codes for the major exo-1,3-beta-glucanase of the cell wall. However, enological performance of most of the strains from the inverse HIP analysis was not improved. In several cases it was rather the opposite (Figure 4, panel A). Indeed strains deleted for *PAF1*, *CTR9*, and *HTL1*, were all impaired for growth in synthetic must, either evaluated after 24 hours or after 21 days (Figure 4, panel A). The implication of these results for the interpretation of inverse HIP and HOP analyses will be discussed below (see Discussion).

According to shared genes, GO terms enriched among the 125 genes identified as haploproficient under Phase II conditions (Workbook S1) were distributed in just two groups (Table S3). Genes included in the first group are related with control of transcription by RNA polymerase II, while the second group is related with hexose metabolism and gluconeogenesis. Again, no clear improvement for growth in synthetic must was observed for 24 hours, and no significant differences were observed for 21 days of fermentation, In contrast, at least two out of the six chosen strains were impaired for growth in synthetic must in a statistically significant way (Figure 4, panel B).

### Inverse HOP analysis: Homozygous deletions conferring a growth advantage in synthetic must

GO terms enriched among the 114 strains identified by the inverse HOP analysis in Phase I are mainly related to peroxisome biogenesis, regulation of phosphorylation, and cell wall biogenesis (Table S4). On the other side, inverse HOP analysis for Phase II identified a set of 92 gene deletions. The main GO terms enriched in this gene set are cytoplasmic translation, mostly involving genes coding for ribosomal proteins, and regulation of cell size (Table S5). Five genes from the top of each list (Phase I and Phase II) were chosen for enological characterization (Figure S4). None of these deletions seemed to confer growth advantage during batch cultivation in synthetic must, rather the opposite. Most of the selected strains showed impaired growth after 24 hours in synthetic must, and some of them, especially from Phase I, also by the end of the fermentation.

### Enological relevance of other gene deletions

The several instances of lack of agreement between results of the inverse HIP and HOP analyses and the phenotypes of isolated strains in synthetic must was intriguing. Also noticeable was that genes involved in tolerance to osmotic stress did not show up in the fitness analysis. To address these questions, gene deletions that had recently been shown to result in fitness advantage or disadvantage under winemaking conditions were tested for enological performance. Despite the differences in the experimental setup (Table S1) we choose the study by Piggott and co-workers [27] due to its close resemblance in media composition. Some strains considered as relevant in the conclusions reached by these authors were selected. In addition, homozygous deletions for *HOG1* and *HOT1*, involved in osmotic stress tolerance, were included in these trials.

**Figure 5:**
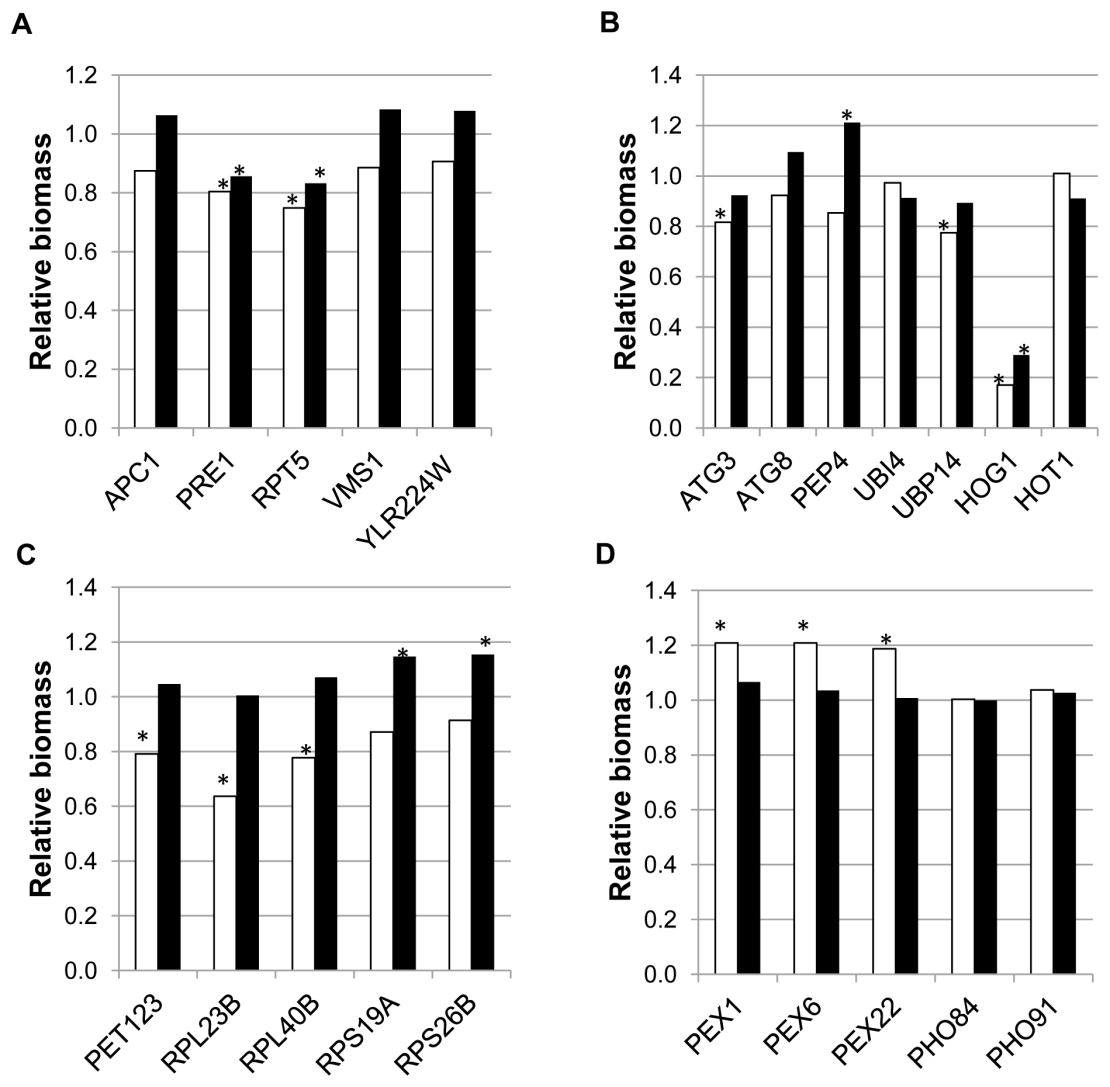
Phenotypic characterization of deletion strains highlighted in Piggott et al [27], as well as *HOG1* and *HOT1* homozygous deletions (manually selected). Detailed legend: Relative biomass after 24 h (white bars) or after 21 days (black bars) for strains deleted for *HOG1* and *HOT1* and strains selected from [27]. Panels A and B: strains selected as showing fitness deficiency. Panels C and D: strains selected as showing fitness advantage. Panels A and C: heterozygous deletions. Panels B and D: homozygous deletions. Relative biomass after 24 h was estimated by comparing turbidity of the indicated strains with the control strain (BY4743) in the same batch. It was measured as NTUs with a 2100N Turbidimeter (HASH, Loveland. CO). Relative biomass after 21 days was estimated by measuring OD_600_ of the homogenized culture and comparing data from the deleted strains with those of the control strain (BY4743) in the same batch. Stars indicate statistically significant differences between BY4743 and the deleted strain.

Selected strains showing fitness deficiencies in the study by Piggott and co-workers [27], showed the expected phenotype for growth in synthetic must during the first 24 hours (Figure 5, panel A and B). In the case of homozygous deletions, growth impairment was less pronounced (with only two strains per analysis, HIP or HOP, showing statistically significant growth impairment) than for strains identified under Phase I conditions (Figure 3, panels A and C), but more than for strains identified under Phase II conditions (Figure 3, panel B).

However, none of the strains selected as haploproficient from the work by Piggott and co-workers [27] seemed to grow better in synthetic must for 24 hours (Figure 5, panel C), although final biomass (21 days) was generally equal or higher than for the wild type. Since all these strains were deleted for genes coding for ribosomal proteins, it seems that decreasing protein biosynthesis is not advantageous for yeast cells during the initial stages of wine fermentation. This makes sense, since this process is required for cell proliferation. However, decreased protein synthesis might provide some advantage for survival in the long term, as indicated by Piggott and co-workers [27], and in agreement with our results on final biomass production (21 days).

In contrast, three out of five strains from the study by Piggott and co-workers [27] clearly showed improved growth compared to BY4743 (24 hours in synthetic must), while two of them were neutral (Figure 5, panel D). After 21 days total biomass was similar to the wild type in all cases. Genes deleted in the three improved strains are involved in peroxisome biogenesis, including *PEX1*, also identified in the inverse HOP analysis for Phase I. These authors linked fitness advantage of these homozygous deleted strains to pexophagy, a specialized form of autophagy induced upon shift from oxidative to fermentative growth. Nevertheless, not all *PEX* genes seem to confer advantage when deleted; since strains deleted for *PEX4* and *PEX10*, also highlighted by the inverse HOP analysis for Phase I, did not show any clear improvement when their enological performance was tested (data not shown).

Concerning genes involved in osmotic stress tolerance, the homozygous strain deleted for *HOT1* behaves similar to BY4743 after 24 hours, and seems to be only slightly impaired for biomass production after 21 days (Figure 5, panel B). However, *HOG1* deletion results in a clear impairment for growth in synthetic must, both in the short and the long term (Figure 5, panel B). Although none of these genes were highlighted in the present HIP and HOP analyses, *HOG1* was close but below the threshold values for direct HOP analysis under Phase I growth conditions. Despite the strong phenotypic effect we have shown for *HOG1* deletion in winemaking conditions, its relevance was also missed in similar competition studies previously published.

## Discussion

This work aimed to identify genes that are targets of the stress factors active during the proliferative stages of wine fermentation (direct HIP analysis) as well as genes required for optimal fitness under these culture conditions (direct HOP analysis). In addition, and according to work published by other authors, it was expected to identify gene deletions, either in homozygosis or heterozygosis, that confer growth advantage in winemaking conditions. The experimental design was decided considering that, under industrial conditions, environmental factors are in constant transformation throughout each fermentation batch. Accordingly, genetic requirements for optimal fitness would be expected to be different in different moments of the fermentation process. The continuous culture strategy adopted in this work was intended to emulate two discrete moments of the fermentation process in which cell proliferation is still relevant. By focusing on the proliferative stages of the fermentation process this work is complementary to that by Piggott and co-workers [27]. In that work, a time-course analysis of batch fermentation of synthetic must was performed. Since most samples were taken in the non-proliferative stages of the fermentation process, and were enriched for viable cells by growth on YPD prior to DNA extraction, this analysis allowed to identify genes required for survival under advanced fermentation conditions. In contrast, the present analysis was expected to identify genes whose activity is required for cell proliferation during the early moments of the fermentation process [26], independently of the effect of its deletion in later fermentation stages. Focusing on the early stages of the fermentation process is also interesting from a practical point of view, since biomass production during the first hours would determine the kinetics of the whole process [28]. Broadly speaking this approach is similar to the HIP analysis performed by Delneri and co-workers [14], using a commercial grape must in continuous culture. But besides including HOP analysis, there are important differences between both studies concerning must composition and oxygen availability, which allowed us to reach steady states with a closer resemblance to specific moments of industrial wine fermentation.

We tried to assess the enological relevance of the results of this fitness analysis by phenotypic characterization of selected strains in synthetic must. A reasonable number of confirmatory results was obtained for the direct analyses, taking as positive results those in which yeast growth in synthetic must was clearly affected for the deleted strain identified by fitness analysis. This was especially clear in the case of direct HOP analysis for Phase I. As a general rule, the observed growth impairment in synthetic must was less severe for heterozygous deleted strains. Indeed, the fitness reduction values shown in Workbook S1 for heterozygous strains were obtained from competition experiments lasting for 20 generations, while those for homozygous strains required only 10 generations to reach similar fitness values.

In contrast, many strains selected from the inverse analyses can be regarded as possible false positives, and very limited discussion of these data will follow. In principle, for any growth condition, the number of genes showing haploproficiency, or conferring growth advantage when deleted, would be expected to be low. The advantage conferred by a gene deletion would be expected to be restricted to a narrow set of culture conditions. While the use of continuous culture allowed us to focus on the proliferative stages of wine fermentation, the fact that we were mimicking two discrete points of the growth curve might be responsible for the lack of agreement with results obtained from cultures spanning for several hours or even days. Also, as suggested by Delneri and co-workers [14], the possibility to identify proficient phenotypes would be particular for competitions below maximal growth rate. This would be the case in continuous culture, as used in the present fitness analysis, despite the relatively high dilution rate employed for the emulation of Phase I conditions, but not for the batch fermentation assays used to investigate enological relevance of the genes identified by fitness analysis.

We have seen that results from competition analyses are not exhaustive, as illustrated by *HOG1*, a gene that was not identified as relevant for fitness under fermentation conditions in either our study or previous related works [14,27], despite the strong impairment we have shown for growth in synthetic must for the corresponding homozygous deleted strain.

In agreement with other works, we found few coincidences between genes simultaneously highlighted by HIP and HOP analyses under the same culture conditions. And almost no overlapping was observed between genes identified as relevant for either Phase I or Phase II of wine fermentation (Figure S1). The fact that genes required for fitness in both growth conditions are so different, and also different from those required for survival in stationary phase [27], somehow confirms one of the hypotheses behind our experimental design: adaptation to the quickly changing growth conditions during grape must fermentation would require the function of different gene sets in different moments of the process. Similarly, gene deletions leading to haploinsufficiency show little overlapping between both fermentation stages. Also as in other works [27], we identified fermentation phenotypes for globally more than 150 ORFs labeled as uncharacterized or dubious in YeastMine [33], ranging from 14% to 26% of the ORFs, depending on the particular dataset (Workbook S1). Assignment of a specific function to these ORFs would require further and targeted research.

Results of the HIP study indicate that transport processes, mainly those involving macromolecules, are the main targets of the stress factors present under Phase I conditions (Table 1). Several functions related with purine metabolism are also targets of stress factors present in Phase I. However this effect might have been amplified by the fact that all strains in the deletion collection carry the *ura3Δ0*/*ura3Δ0* marker. It is also worth mentioning that two proteins involved in sugar signaling (encoded by *RGT2* and *SNF3*) seem also to be affected by the stress conditions in Phase I, and their enological relevance was also shown by the phenotype of the isolated strains. Targets for the stress conditions in Phase II seem to be more diverse, and only a few genes contribute to the GO term enrichment shown in Table 2. Lipid metabolism and cell wall biosynthesis appear as the most clearly enriched categories under these culture conditions.

The auxotrophic nature of some of the genes identified in the direct HOP analysis of Phase I indicated that some nutrients might be in short supply in our synthetic must. This was confirmed to be also the case for at least adenine and lysine in a natural white grape must. On the contrary, inositol and serine deficiencies seem to be specific for the synthetic must recipe used in this work, as it was not confirmed in natural grape must. This result is in agreement with the recent findings on nutritional requirements of the BY-strains [31]. These nutritional requirements were not identified in Phase II analysis, and since osmotic pressure is high in both conditions, this difference must be attributed to differences on the abundance and nature of the nitrogen source, and mainly to a different growth rate. Similar to us, the HIP analysis by Delneri and co-workers [14] did not identify nutritional requirements for yeast growth in grape must, but their conclusion that grape must provide all the amino acids in sufficient amounts for yeast growth (but not pirimidines) is not sustained by our direct HOP analysis in Phase I, at least for some amino acids. Indeed, using heterozygous strains [14], does not seem to be the best tool to identify nutritional requirements. Knowing that adenine and lysine are in short supply in at least some natural grape musts might be relevant for the design of nitrogen supplements for winemaking.

Leaving apart nutritional requirements, genes necessary for optimal fitness in Phase I (as deduced from the direct HOP analysis) seem to be falling in many diverse categories, so that just a few of them contribute to enriched GO terms. These genes are mainly related to nucleic acid dynamics, like transcription, replication, and chromatin assembly (Table 4). On the other side, and according to the GO enrichment analysis shown in Table 5, optimal fitness under Phase II conditions seems to require proper functioning of localization processes. Specifically, transport phenomena related to vacuolar function (*ATG* and *VPS* genes, for example), appear enriched in this analysis. Piggott and co-workers [27] also showed autophagic functions to be required for survival under winemaking conditions, even though co-occurrence of specific *ATG* or *VPS* genes in both studies is low. Other cellular functions also clearly highlighted in this analysis for Phase II are protein folding and chromatin dynamics in relation with carbon catabolite repression (Table 5).

## Conclusions

Cell functions required for optimal fitness, as well as the targets for stress factors present under the growth conditions analyzed in this study are highly diverse. This would be in agreement with the also diverse set of stress conditions acting on yeast cells under winemaking conditions. One of the hypotheses behind our experimental design was confirmed: adaptation to the quickly changing growth conditions during grape must fermentation requires the function of different gene sets in different moments of the process. Readers interested in specific genes are encouraged to check Workbook S1. The contribution of most genes to fitness at the individual level would be relatively low, or else, the most relevant pathways might be genetically redundant, and difficult to identify by the analysis of strains deleted for single genes.

Despite these considerations, it can be said that transport processes, especially those involving macromolecules, seem to be one of the main targets of some of the stress factors found by yeast cells during the early stages of wine fermentation. Also relevant is the fact that some proteins involved in sugar signaling are negatively affected by these environmental conditions. Some aspects of lipid metabolism seem to be also affected in later stages (Phase II) of yeast growth in synthetic must. Direct HOP analysis under Phase I conditions highlighted the importance of proper nucleic acid dynamics for optimal fitness under these stressful conditions. Vacuolar functions appear as especially relevant for survival during Phase II, and according to Piggott and co-workers [27] for survival until the end of the fermentation process. We have also confirmed the advantage for growth under enological conditions conferred by the deletion of some *PEX* genes, as previously observed [27]. In addition, we concluded that reduced cell content for some ribosomal proteins was not advantageous (rather the opposite) for yeast cells during the early stages of the fermentation process, in spite of a possible advantage for survival in advanced stages, as found by other authors. Finally, our study has found adenine and lysine to be in short supply in at least some natural musts. This information might be interesting for the development of yeast nutrients for wine fermentation. Further research would be required to enrich this and previous works, and to help completing the picture of genes relevant for yeast performance under winemaking conditions.

## Supporting Information

Table S1 **Growth and sampling strategies in different HIP HOP studies under winemaking-like conditions.**
(DOCX)

Table S2 **Gene Ontology enrichment analysis for genes identified by inverse HIP analysis under Phase I fermentation conditions.** Unedited results of the GO enrichment analysis are shown in workbook S2.
(DOCX)

Table S3 **Gene Ontology enrichment analysis for genes identified by inverse HIP analysis under Phase II fermentation conditions.** Unedited results of the GO enrichment analysis are shown in workbook S2.
(DOCX)

Table S4 **Gene Ontology enrichment analysis for genes identified under inverse HOP analysis under Phase I fermentation conditions.** Unedited results of the GO enrichment analysis are shown in workbook S2.
(DOCX)

Table S5 **Gene Ontology enrichment analysis for genes identified by inverse HOP analysis under Phase II fermentation conditions.** Unedited results of the GO enrichment analysis are shown in workbook S2.
(DOCX)

Figure S1 **Shared genes among those highlighted in different analyses and fermentation Phases.**
(PPTX)

Figure S2 **Relative biomass observed after 24 h (white bars) or after fermentation arrest (black bars) for strains identified simultaneously under Phase I and Phase II conditions by HIP analyses.** Relative biomass after 24 h was estimated by comparing turbidity of the indicated strains with the control strain (BY4743) in the same batch. It was measured as NTUs with a 2100N Turbidimeter (HASH, Loveland. CO). Relative biomass after 21 days was estimated by measuring OD600 of the homogenized culture and comparing data from the deleted strains with those of the control strain (BY4743) in the same batch. No statistically significant differences were found between BY4743 and the deleted strain.
(PPTX)

Figure S3 **Relative biomass observed after 24 h (white bars) or after fermentation arrest (black bars) for strains identified simultaneously under Phase I and Phase II conditions by HOP analyses.** Relative biomass after 24 h was estimated by comparing turbidity of the indicated strains with the control strain (BY4743) in the same batch. It was measured as NTUs with a 2100N Turbidimeter (HASH, Loveland. CO). Relative biomass after 21 days was estimated by measuring OD600 of the homogenized culture and comparing data from the deleted strains with those of the control strain (BY4743) in the same batch. Stars indicate statistically significant differences between BY4743 and the deleted strain.
(PPTX)

Figure S4 **Relative biomass observed after 24 h (white bars) or after fermentation arrest (black bars) for strains identified under Phase I (panel **A**) and Phase II (panel **B**) conditions by inverse HOP analyses.** Relative biomass after 24 h was estimated by comparing turbidity of the indicated strains with the control strain (BY4743) in the same batch. It was measured as NTUs with a 2100N Turbidimeter (HASH, Loveland. CO). Relative biomass after 21 days was estimated by measuring OD600 of the homogenized culture and comparing data from the deleted strains with those of the control strain (BY4743) in the same batch. Stars indicate statistically significant differences between BY4743 and the deleted strain.
(PPTX)

Workbook S1 **Complete gene lists issued from fitness analysis.**
(XLSX)

Workbook S2 **Unedited results of the GO enrichment analysis.**
(XLSX)
