# Supplementary material for: Genome-Wide Study of the Adaptation of Saccharomyces cerevisiae to the Early Stages of Wine Fermentation
Source: PLoS One. 2013 Sep 5;8(9):e74086. doi: 10.1371/journal.pone.0074086 (PMC3764036; doi:10.1371/journal.pone.0074086)
Supplement: Table S1 — Growth and sampling strategies in different HIP HOP studies under winemaking-like conditions. (DOCX) [file pone.0074086.s001.docx]

**Table S1.** Growth and sampling strategies in different HIP HOP studies under winemaking-like conditions.

|  | This study | Piggot et al., 2011 | Delneri et al., 2008 |
| --- | --- | --- | --- |
| Hexose content | 200 g/L | 200 g/L | 100 g/L |
| Nitrogen source | Mixed ^a^ | Ammonium | Mixed ^b^ |
| Generations | 20/10^c^ | Batch | 35 |
| Dilution rate (h^-1^) | 0.10/0.23^d^ | None | 0.1 |
| Pre-DNA extraction treatment | None | YPD | None |
| *URA3* supplementation | Uridine | Uracil | None^b^ |
| Aeration | Nitrogen | Fermentation lock | Aerobic |
| Biological replicates | 3 | 1(time-course) | 2 |
| Collection | Both | Both | Heterozygous |
| Unstressed control condition | YPD  and t=24 h | t=24 h | t=24 h |

^a^ The mix of nitrogen sources in the pressed white grape juice corresponded to 79.5 mg N/l in the form of amino acids and 122.5 mg N/l in the form of ammonium.

^b^ In the study of winemaking-like conditions, Delneri et al. directly used 100% pure pressed white grape juice (Sunpride), with no further additions.

^c^ Continuous growth cultures were maintained until reach 20 generations for Heterozygous collection and 10 generation for Homozygous collection.

^d^ 0.23 h^-1^ for the simulation of Phase I and 0.1 h^-1^ for the simulation of Phase II conditions.
