# Supplementary material for: Genome-Wide Study of the Adaptation of Saccharomyces cerevisiae to the Early Stages of Wine Fermentation
Source: PLoS One. 2013 Sep 5;8(9):e74086. doi: 10.1371/journal.pone.0074086 (PMC3764036; doi:10.1371/journal.pone.0074086)
Supplement: Table S2 — Gene Ontology enrichment analysis for genes identified by inverse HIP analysis under Phase I fermentation conditions. Unedited results of the GO enrichment analysis are shown in workbook S2. (DOCX) [file pone.0074086.s002.docx]

**Table S2.** Gene Ontology enrichment analysis for genes identified by inverse HIP analysis under Phase I fermentation conditions. Unedited results of the GO enrichment analysis are shown in supplementary workbook S2.

| **GO Term** | **p-value** | **#^a^** | **Genes in group^b^** |
| --- | --- | --- | --- |
| peptidyl-lysine methylation [GO:0018022] | 9E-06 | 4 | AHC2, CTR9, DBP3, EFM2, HTL1, PAF1, RSC6, RSP5, SEE1 |
| positive regulation of histone H3-K36 methylation [GO:0000416/0000416/0097198/2001253/2001255] | 0.0003 | 2 | s |
| peptidyl-lysine modification [GO:0018205] | 0.00055 | 5 | s |
| snoRNA transcription from an RNA polymerase II promoter [GO:0001015/0071619/1901407/1901409/2001163/2001165] | 0.0006 | 2 | s |
| protein methylation [GO:0006479] | 0.00115 | 4 | s |
| histone H3-K36 methylation [GO:0000414/0009302/0031062/0051569  /2001166/2001173/0008213/0010452/0018023/0033182/0033523/0071894] | 0.00149 | 2 | s |
| regulation of transcription-coupled nucleotide-excision repair [GO:0090262/2000819/2001207/2001209] | 0.00207 | 2 | s |
| regulation of histone methylation [GO:0031060] | 0.00274 | 2 | s |
| G1 phase of mitotic cell cycle [GO:0000080/0032786/0051318] | 0.00303 | 3 | s |
| regulation of DNA-dependent transcription, elongation [GO:0032784] | 0.00403 | 3 | s |
| chromatin organization [GO:0006325] | 0.00404 | 8 | s |
| regulation of DNA repair [GO:0006282/0006362/0031058/0044273] | 0.00435 | 2 | s |
| glutathione catabolic process [GO:0006751/0043171] | 0.00274 | 2 | DUG1, DUG2 |
| regulation of response to DNA damage stimulus [GO:2001020] | 0.00042 | 3 | AGA1, ATG15, CTR9, DPB3, GPG1, HCM1, HOF1, HTL1, MSH2, PAF1, PSY4, PTC6, RAD18, RSP5, SDS3, SPH1, SRB8, WHI2, WSC3 |
| regulation of chromatin silencing at telomere [GO:0031938] | 0.00246 | 3 | s |
| postreplication repair [GO:0006301] | 0.00246 | 3 | s |
| cellular response to stimulus [GO:0051716] | 0.00439 | 17 | s |
| autophagy [GO:0006914] | 0.0046 | 5 | s |
| error-prone translesion synthesis [GO:0042276] | 0.00529 | 2 | s |
| regulation of transcription involved in G1 phase of mitotic cell cycle [GO:0000114] | 0.0074 | 2 | s |
| regulation of chromatin silencing [GO:0031935/0060968/0080135] | 0.00758 | 3 | s |
| histone ubiquitination [GO:0016574/0019985/0045910] | 0.00858 | 2 | s |
| cell cycle [GO:0007049] | 0.00526 | 14 | BNI5, BUD20, CSM1, CTR9, HCM1, HOF1, HTL1, MSH2, PAF1, PSY4, RSC6, SAP185, SPH1, WHI2 |
| interphase of mitotic cell cycle [GO:0051329/ GO:0051325] | 0.00906 | 5 | s |
| phosphate ion transport [GO:0006817] | 0.0074 | 2 | PHO87, PHO89 |

a: number of genes shared with its group

b: s=same as above
