# Supplementary material for: Genome-Wide Study of the Adaptation of Saccharomyces cerevisiae to the Early Stages of Wine Fermentation
Source: PLoS One. 2013 Sep 5;8(9):e74086. doi: 10.1371/journal.pone.0074086 (PMC3764036; doi:10.1371/journal.pone.0074086)
Supplement: Table S3 — Gene Ontology enrichment analysis for genes identified by inverse HIP analysis under Phase II fermentation conditions. Unedited results of the GO enrichment analysis are shown in workbook S2. (DOCX) [file pone.0074086.s003.docx]

**Table S3.** Gene Ontology enrichment analysis for genes identified by inverse HIP analysis under Phase II fermentation conditions. Unedited results of the GO enrichment analysis are shown in supplementary workbook S2.

| GO Term | p-value | #^a^ | Genes in group^b^ |
| --- | --- | --- | --- |
| regulation of protein complex assembly [GO:0043254] | 0.000232 | 6 | BUD14, CAP2, CHD1, FKH1, MVB12, RPT3, RPT6, SPO22, TAF10, TAF6 |
| regulation of cellular component biogenesis [GO:0044087] | 0.0003 | 7 | s |
| negative regulation of protein complex assembly [GO:0031333] | 0.000567 | 3 | s |
| regulation of transcription initiation from RNA polymerase II promoter [GO:0060260/2000142] | 0.002451 | 3 | s |
| transcription initiation from RNA polymerase II promoter [GO:0006367] | 0.002553 | 5 | s |
| regulation of sequence-specific DNA binding transcription factor activity [GO:0051090] | 0.00077 | 3 | s |
| RNA polymerase II transcriptional preinitiation complex assembly [GO:0051123] | 0.006403 | 4 | s |
| DNA-dependent transcription, initiation [GO:0006352] | 0.006404 | 5 | s |
| DNA-dependent transcriptional preinitiation complex assembly [GO:0070897] | 0.008777 | 4 | s |
| negative regulation of protein polymerization [GO:0032272] | 0.007892 | 2 | s |
| regulation of carbohydrate biosynthetic process [GO:0043255] | 0.001297 | 4 | FYV10, PDA1, PIG2, PKP2, SKT5, SNF4, TPI1, VID28 |
| glucose metabolic process [GO:0006006] | 0.001695 | 7 | s |
| regulation of gluconeogenesis [GO:0006111] | 0.002017 | 3 | s |
| gluconeogenesis [GO:0006094/0010906] | 0.002425 | 4 | s |
| hexose biosynthetic process [GO:0019319] | 0.002712 | 4 | s |
| monosaccharide biosynthetic process [GO:0046364] | 0.003021 | 4 | s |
| hexose metabolic process [GO:0019318] | 0.003753 | 7 | s |
| regulation of cellular carbohydrate metabolic process [GO:0010675/0006109] | 0.004934 | 4 | s |
| monosaccharide metabolic process [GO:0005996] | 0.005198 | 7 | s |
| carbohydrate biosynthetic process [GO:0016051] | 0.006042 | 6 | s |

a: number of genes shared with its group

b: s=same as above
