# Supplementary material for: Genome-Wide Study of the Adaptation of Saccharomyces cerevisiae to the Early Stages of Wine Fermentation
Source: PLoS One. 2013 Sep 5;8(9):e74086. doi: 10.1371/journal.pone.0074086 (PMC3764036; doi:10.1371/journal.pone.0074086)
Supplement: Table S4 — Gene Ontology enrichment analysis for genes identified under inverse HOP analysis under Phase I fermentation conditions. Unedited results of the GO enrichment analysis are shown in workbook S2. (DOCX) [file pone.0074086.s004.docx]

**Table S4.** Gene Ontology enrichment analysis for genes identified under inverse HOP analysis under Phase I fermentation conditions. Unedited results of the GO enrichment analysis are shown in supplementary workbook S2.

| **GO Term** | **p-value** | **#^a^** | **Genes in group^b^** |
| --- | --- | --- | --- |
| protein import into peroxisome matrix [GO:0016558] | 1.74E-05 | 5 | ATG36, FIS1, PEX1, PEX10, PEX12, PEX15, PEX4, SNF8, STP22, VPS27, VPS36 |
| protein targeting to peroxisome [GO:0006625/0043574/0072662/0072663] | 3.5E-05 | 5 | s |
| receptor recycling [GO:0001881/0016562/0043112] | 4.15E-05 | 3 | s |
| peroxisome organization [GO:0007031] | 0.00013 | 7 | s |
| intracellular protein transmembrane import [GO:0044743] | 0.00457 | 5 | s |
| establishment of protein localization to organelle [GO:0072594] | 0.00866 | 9 | s |
| ubiquitin-dependent protein catabolic process via the multivesicular body sorting pathway [GO:0043162] | 0.00039 | 4 | s |
| positive regulation of kinase activity [GO:0033674/0051347] | 0.00108 | 3 | ASG1, BRE5, CIN5, DOT6, ETT1, FRE1, GAT2, GPB1, IKI3, IRS4, MAC1, MAL33, MPD2, NDJ1, NPR1, PBP1, PCA1, PEX1, PEX15, PEX4, PFK26, PIN4, RAS2, RFM1, RPI1, SAP190, SNF8, SPT2, SSK1, SSK2, STB1, SWE1, TIF2, TIP41, UBC13, URA2, VAC14, VPS27, VPS36 |
| osmosensory signaling via phosphorelay pathway [GO:0007234] | 0.00156 | 2 | s |
| positive regulation of phosphorus metabolic process [GO:0010562/0045937] | 0.0017 | 4 | s |
| regulation of signaling [GO:0023051] | 0.00236 | 7 | s |
| regulation of biological process [GO:0050789] | 0.0037 | 36 | s |
| phosphorelay signal transduction system [GO:0000160] | 0.00381 | 2 | s |
| positive regulation of phosphorylation [GO:0042327] | 0.00396 | 3 | s |
| regulation of phosphorylation [GO:0042325] | 0.00487 | 5 | s |
| protein polyubiquitination [GO:0000209] | 0.00522 | 3 | s |
| biological regulation [GO:0065007] | 0.00527 | 39 | s |
| glutathione catabolic process [GO:0006751] | 0.00257 | 2 | DUG1, DUG2 |
| peptide catabolic process [GO:0043171] | 0.00696 | 2 | s |
| cell wall organization or biogenesis [GO:0071554] | 0.00354 | 9 | BPH1, CHS6, DCW1, IRS4, KNH1, KRE1, PUN1, RPI1, UBC7 |
| fungal-type cell wall organization or biogenesis [GO:0071852] | 0.00533 | 8 | s |
| tRNA wobble base modification [GO:0002097/0002098] | 0.0084 | 3 | IKI3, SAP190, TUM1 |

a: number of genes shared with its group

b: s=same as above
