# Supplementary material for: Genome-Wide Study of the Adaptation of Saccharomyces cerevisiae to the Early Stages of Wine Fermentation
Source: PLoS One. 2013 Sep 5;8(9):e74086. doi: 10.1371/journal.pone.0074086 (PMC3764036; doi:10.1371/journal.pone.0074086)
Supplement: Table S5 — Gene Ontology enrichment analysis for genes identified by inverse HOP analysis under Phase II fermentation conditions. Unedited results of the GO enrichment analysis are shown in workbook S2. (DOCX) [file pone.0074086.s005.docx]

**Table S5.** Gene Ontology enrichment analysis for genes identified by inverse HOP analysis under Phase II fermentation conditions. Unedited results of the GO enrichment analysis are shown in supplementary workbook S2.

| **GO Term** | **p-value** | **#^a^** | **Genes in group^b^** |
| --- | --- | --- | --- |
| cytoplasmic translation [GO:0002181] | 0.00066 | 9 | RPL2A, RPL8A, RPS0A, RPS10B, RPS1A, RPS4B, RPS6B, RPS8A, SRO9 |
| histone deubiquitination [GO:0016578] | 0.00117 | 2 | UBP8, SGF11 |
| fructose metabolic process [GO:0006000] | 0.00287 | 2 | HXK2, PFK26 |
| regulation of cellular component size [GO:0032535/0090066] | 0.0039 | 4 | BNR1, HXK2, RPA14, SWE1 |
| regulation of cell size [GO:0008361] | 0.00847 | 3 | s |
| apoptotic process [GO:0006915/0008219/0012501/0016265] | 0.0077 | 3 | FIS1, FYV10, OYE2 |

a: number of genes shared with its group

b: s=same as above
