# Supplementary material for: Genome-Wide Study of the Adaptation of Saccharomyces cerevisiae to the Early Stages of Wine Fermentation
Source: PLoS One. 2013 Sep 5;8(9):e74086. doi: 10.1371/journal.pone.0074086 (PMC3764036; doi:10.1371/journal.pone.0074086)
Supplement: Figure S1 — Shared genes among those highlighted in different analyses and fermentation Phases. (PPTX) [file pone.0074086.s006.pptx]

## Slide 1
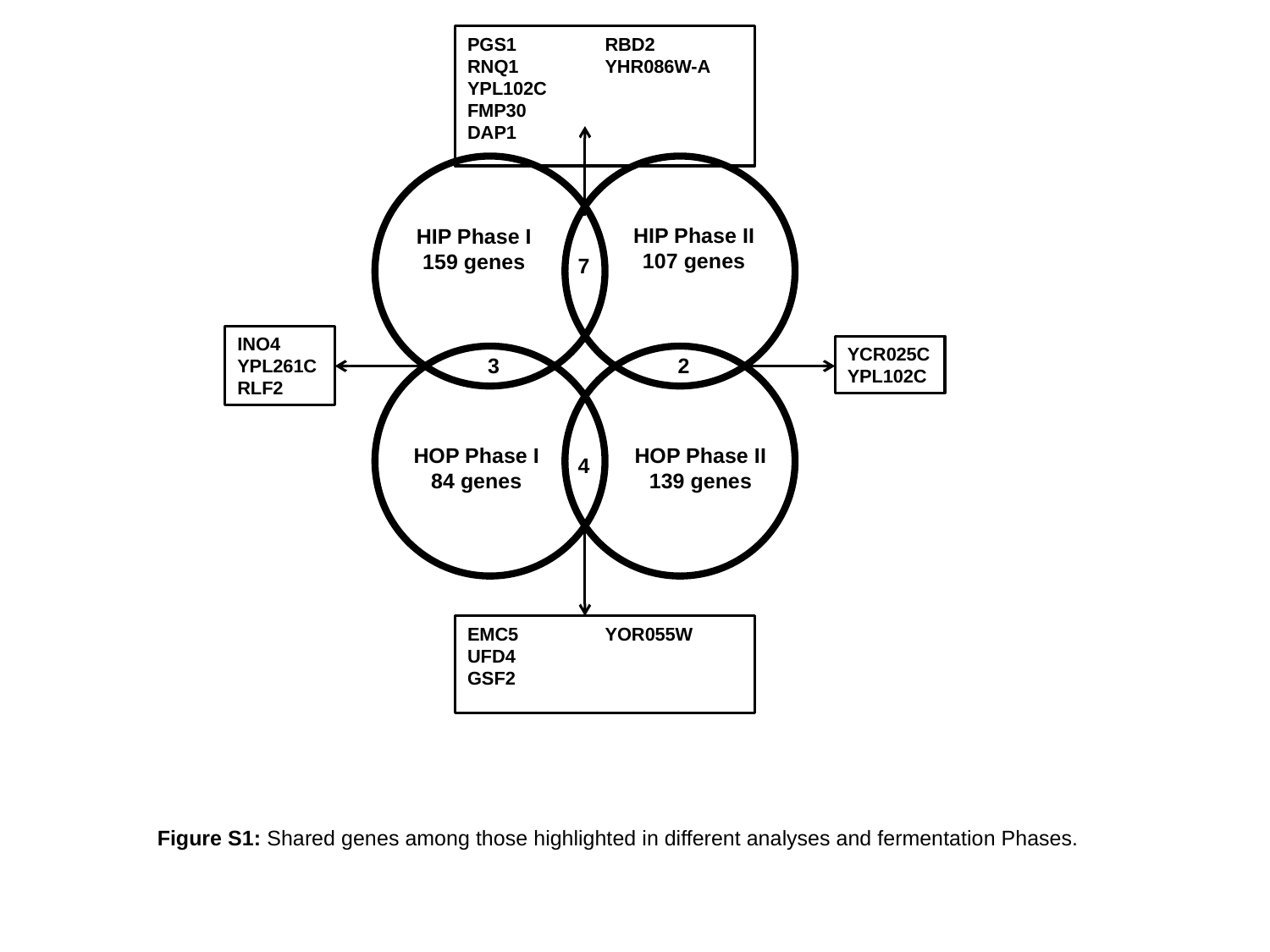

PGS1
RNQ1
YPL102C
FMP30
DAP1
RBD2
YHR086W-A
HIP Phase II
107 genes
HIP Phase I
159 genes
7
INO4
YPL261C
RLF2
YCR025C
YPL102C
3
2
HOP Phase I
84 genes
HOP Phase II
139 genes
4
EMC5
UFD4
GSF2
YOR055W
Figure S1: Shared genes among those highlighted in different analyses and fermentation Phases.
