# Supplementary material for: Genome-Wide Study of the Adaptation of Saccharomyces cerevisiae to the Early Stages of Wine Fermentation
Source: PLoS One. 2013 Sep 5;8(9):e74086. doi: 10.1371/journal.pone.0074086 (PMC3764036; doi:10.1371/journal.pone.0074086)
Supplement: Figure S3 — Relative biomass observed after 24 h (white bars) or after fermentation arrest (black bars) for strains identified simultaneously under Phase I and Phase II conditions by HOP analyses. Relative biomass after 24 h was estimated by comparing turbidity of the indicated strains with the control strain (BY4743) in the same batch. It was measured as NTUs with a 2100N Turbidimeter (HASH, Loveland. CO). Relative biomass after 21 days was estimated by measuring OD600 of the homogenized culture and comparing data from the deleted strains with those of the control strain (BY4743) in the same batch. Stars indicate statistically significant differences between BY4743 and the deleted strain. (PPTX) [file pone.0074086.s008.pptx]

## Slide 1
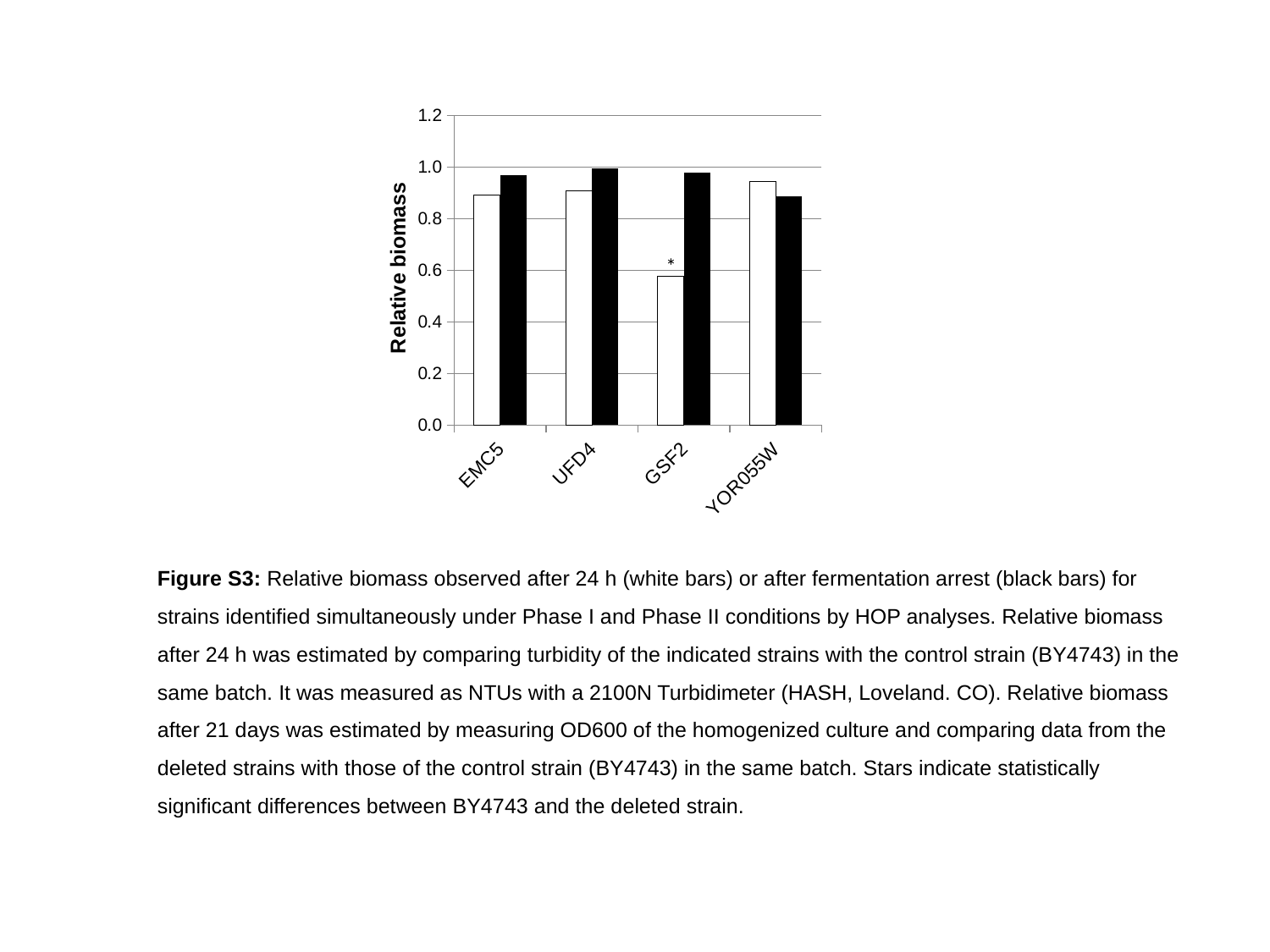

### Chart
| Category | | |
|---|---|---|
| EMC5 | 0.8936170212765964 | 0.9680397727272727 |
| UFD4 | 0.9095744680851064 | 0.9950284090909092 |
| GSF2 | 0.5757978723404276 | 0.9786931818181823 |
| YOR055W | 0.9441489361702138 | 0.8877840909090917 |*
Figure S3: Relative biomass observed after 24 h (white bars) or after fermentation arrest (black bars) for strains identified simultaneously under Phase I and Phase II conditions by HOP analyses. Relative biomass after 24 h was estimated by comparing turbidity of the indicated strains with the control strain (BY4743) in the same batch. It was measured as NTUs with a 2100N Turbidimeter (HASH, Loveland. CO). Relative biomass after 21 days was estimated by measuring OD600 of the homogenized culture and comparing data from the deleted strains with those of the control strain (BY4743) in the same batch. Stars indicate statistically significant differences between BY4743 and the deleted strain.
